# CEBA: A Data Lake for Data Sharing and Environmental Monitoring

**DOI:** 10.3390/s22072733

**Published:** 2022-04-02

**Authors:** David Sarramia, Alexandre Claude, Francis Ogereau, Jérémy Mezhoud, Gilles Mailhot

**Affiliations:** 1 Laboratoire de Physique de Clermont, Université Clermont Auvergne, CNRS/IN2P3, 63000 Clermont-Ferrand, France; alexandre.claude@clermont.in2p3.fr; 2 Mésocentre, DSI, Projet I-Site CAP 20-25, Université Clermont Auvergne, 63000 Clermont-Ferrand, France; francis.ogereau@uca.fr (F.O.); jeremy.mezhoud@uca.fr (J.M.); 3 Institut de Chimie de Clermont-Ferrand, Université Clermont Auvergne, CNRS, Clermont Auvergne INP, 63000 Clermont-Ferrand, France; gilles.mailhot@uca.fr

**Keywords:** data lake, indexes, data visualization, internet of things, data management, environmental sensors

## Abstract

This article presents a platform for environmental data named “Environmental Cloud for the Benefit of Agriculture” (CEBA). The CEBA should fill the gap of a regional institutional platform to share, search, store and visualize heterogeneous scientific data related to the environment and agricultural researches. One of the main features of this tool is its ease of use and the accessibility of all types of data. To answer the question of data description, a scientific consensus has been established around the qualification of data with at least the information “when” (time), “where” (geographical coordinates) and “what” (metadata). The development of an on-premise solution using the data lake concept to provide a cloud service for end-users with institutional authentication and for open data access has been completed. Compared to other platforms, CEBA fully supports the management of geographic coordinates at every stage of data management. A comprehensive JavaScript Objet Notation (JSON) architecture has been designed, among other things, to facilitate multi-stage data enrichment. Data from the wireless network are queried and accessed in near real-time, using a distributed JSON-based search engine.

## 1. Introduction

Climate change and human activities have an increasing impact on ecosystems [1,2], and the documentation of global change patterns has been identified as a first step to face the changing environment to understand the global loss of biodiversity [3] leading to the need of a quick, reliable access to high-quality data.

It draws the importance of standards in data and metadata, for example in ecology [4] without being completely defined yet. In this domain, even if a number of repositories to store shared data have been created and on going developments are trying to define common data and metadata standards [5], existing data are not exposed in a standard, machine-readable format using a common vocabulary. Common to all fields, this problem has for example been addressed by the biodiversity community with the creation of the Darwin Core [3].

As Deligiannis et al. explain in [6], many stakeholders are lacking the resources for infrastructure and/or computing expertise, and still rely on outdated approaches such as (i) storing their data in spreadsheets or raw files, (ii) sharing their data with colleagues through email, cloud uploads of zip files, or even by snail mailing electronic copies in removable media, and (iii) analyzing the data via sub-standard tools and trial software. They also mentioned that the proposed data storage solutions usually require significant computing infrastructure and need the constant support and active involvement of Information Technology (IT) experts even for trivial tasks such as incorporating new data. Managing the data heterogeneity and interoperability are still common challenges [7]. For example, Ciampittiello et al. [8] identify that the specific needs of research in the environmental field are among others the collection of high-frequency data that are not readily available in commercial instruments, a data processing software and interoperability. As it is the case in many domains, they also indicate that the sensors used are both commercially produced and built by the individual research groups, according to their different specific needs [9], generating even more heterogeneity. The data formats of different sensors are often incompatible, complicating data processing [10]. Thus, Retamar et al. [11] highlight that the sharing, the exchange and the interoperability of environmental information, individual data, or boundary information constitute the basis for an in-depth knowledge of a territory. Hossain et al. [12] argue that data should not be captive by its producers but need to be freely accessible while Shadbolt et al. [13] explain that knowledge [14] and new services can be drown from data.

However, this implies the development of infrastructures such as data repositories allowing data to be shared and valued [15]. Regarding this, ontologies and Findable, Accessible, Interoperable, and Reusable (FAIR) systems are presently able to handle these challenges effectively [16]. As Wilkinson et al. [16] explain in the basis of FAIR principles, there are numerous and diverse stakeholders and have much to gain that researchers wanting to share, obtain credit, and reuse each other’s data and interpretations; funding agencies (private and public) are increasingly concerned with long-term data stewardship; and the data science community is interested in mining, integrating and analyzing new and existing data to advance discovery. According to Murray-Rust [17], full open access is the simplest solution to share and reuse data, and it can also increase the quality of data. This open data movement has its roots in academic circles over the past 50 years [18].

At the same time, Internet of Things (IoT) has become a common technology for large deployment with multiple applications [19] such as smart health care [20], environment monitoring [8,21,22,23], smart cities [24], industrial control [10,25] and agriculture [26,27,28]. The number of connected devices is estimated at the horizon of 2022 at 42.5 billion and at the horizon of 2025 at 75.5 billion with a data volume created by IoT connections projected to reach a massive total of 79.4 zettabytes (https://www.statista.com/statistics/471264/iot-number-of-connected-devices-worldwide/ (accessed on 1 December 2021)).

This kind of systems is well suited for environmental studies and smart agriculture by deploying a monitoring system in a remote area when a high data rate is not required and where minimal power consumption is sufficient [29], making possible a long-term monitoring (see for example [23,30] for environment applications and [26] for smart agriculture application). From the end-user point of view, the IoT seems to make real-time uses possible. Ciampittiello et al. [8] explain, real-time processing requires continual input, constant processing, and steady output of data. For some applications, the near real-time is sufficient, and has become an hot-topic with the widespread adoption of IoT (see for example the applications to smart agriculture [26,31]). In near real-time processing the frequency of transmission is important, but the whole processing time (from acquisition to visualization) can be in minutes (up to 10), particularly if environmental process under study is stable [32]. In the environmental domain, a spatial analysis and representation are commonly implemented [32], by at least georeferencing with GPS WSG84 latitude and longitude combined with an UTC timestamp. A lot of work on the issue of monitoring the environment using wireless technologies have been conducted [33,34,35], but often the solutions proposed are theoretical, on a particular domain or for short term experiments. New telecommunication technologies such Long Range (LoRa (https://lora-alliance.org/ (accessed on 1 December 2021))) or SigFox (https://www.sigfox.com/en (accessed on 1 December 2021)) appliance with low velocity can transmit data over kilometers with small infrastructure and tiny power sources, which perfectly fit the need for large deployment in the environment.

In consequence, a lot of research is conducted on data storage in the cloud, but few works take into account the complete integration of the data from the sensor to the cloud for long term with various types of sensors and on several sites. Nevertheless, there are works on the collection of long-term data in large national data centers or data hubs. In France, such centers are among others Pôle National de Données de Biodiversité (PNDB) (https://www.pndb.fr/pages/fr/portail-pndb (accessed on 1 December 2021)) and data terra (https://www.data-terra.org/en/accueil/ (accessed on 1 December 2021)), which are able to process large amount of data with large resources (human and computational).

Along with the new technologies enabling data analysis, storage and processing architecture play also a key role in the big data ecosystem [7]. Whatever the architectures proposed [26], they aim to facilitate the storing, manipulating, analyzing and accessing for structured and unstructured data [36]. Among those modern architectures, data lakes are of main interest as their design aims to store a large volume of data in any format and structure, and to provide services for data access and analysis [37,38]. They are particularity relevant in the context of environmental monitoring and agriculture as they can manage raw data structured or not. According to Fang [39], a data lake is a cost-efficient data storage system that can improve data analyzing process from ingestion, storage, exploration and exploitation of data in their native format.

In this paper, we present the “Cloud Environnemental au Bénéfice de l’Agriculture” or “An Environmental Cloud for the Benefit of Agriculture” (CEBA) which is one deliverable of the Challenge 1 “Sustainable agroecosystems in a context of global change” of the the Initiatives Science–Innovation–Territories–Economy (I-Site) Clermont CAP 20-25 Projet [40]. By proposing an on-premise data lake, the CEBA aims to fill the gap of a regional tool in Auvergne (France) to store, visualize, analyze and share heterogeneous data (structured or not) collected from research programs and studies related to the environment and agriculture, whether these data come from files, databases or sensor networks with an authentication service and ensuring data ownership.

**Figure 1:**
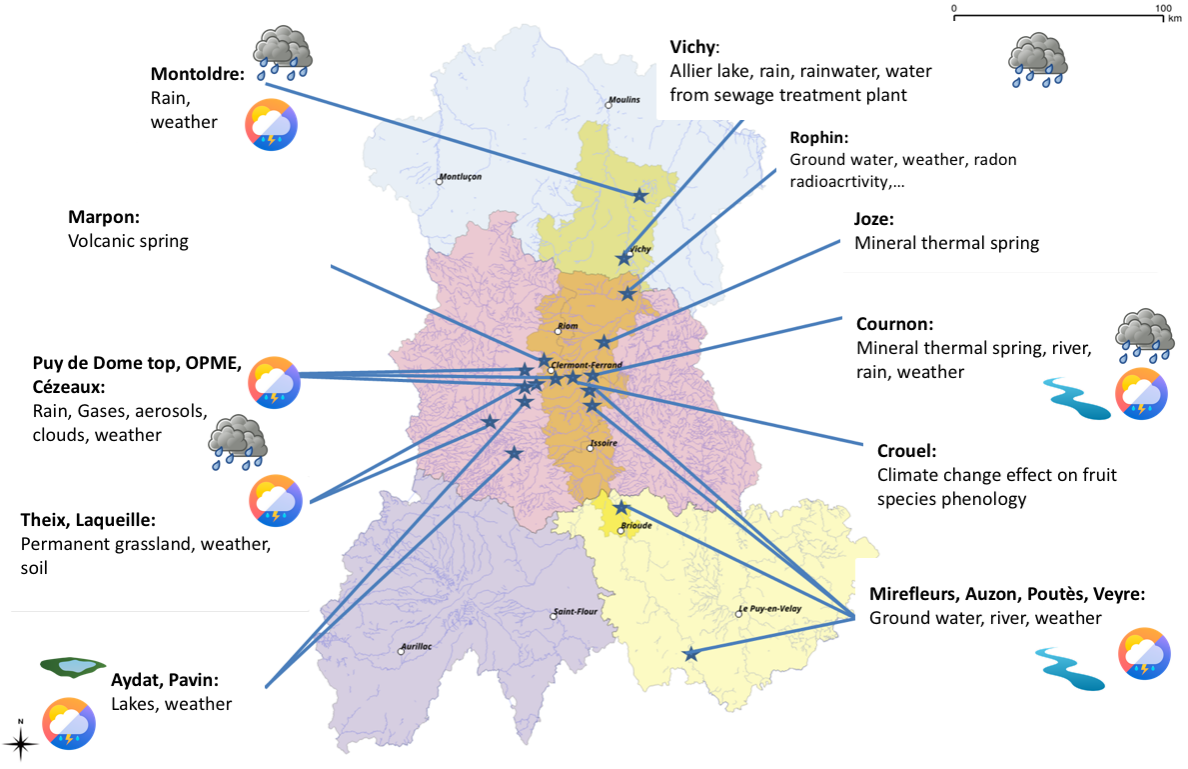
Geographical area and sites covered by the CEBA.

The geographical area covered by the CEBA is the former Auvergne region, with near twenty sites identified covering a large scope of data and analysis to manage (see Figure 1).

The CEBA proposes an innovative architecture for research teams that do not have IT computer staff to (i) record, organize and manage collected data with a metadata description for datafiles up to several gigabyte with a simple normative metadata management; (ii) collect IoT data with free format and structure for low output sensors networks such as LoRa or SigFox; (iii) access and visualize IoT (hot) data in near real-time, access and query historical (cold) data, extract in several formats; (iv) perform bounding box spatial query on data; (v) search for data and dataset in a data catalog according to keywords and geographical GPS WSG84 coordinates; (vi) share whole datasets and datafile with authenticated users and public; (vii) perform basic and advanced user management tasks (such as user permissions, data and metadata access).

The contributions of the work performed in the CEBA is an innovative on-premise data lake with the following features: a data catalogue managing spatially referenced resources, an ingestion platform to collect IoT data regardless of its format and structure, a storage platform to upload and store any type of data files with query solutions, a visualization platform to access hot data in near real-time, some spatial and temporal query functionalities. To achieve these objectives, an efficient storage architecture and data management pipeline are proposed to provide a robust, scalable and extensible solution. In addition, these elements can allow data enrichment for sensor networks or if some metadata are lacking. All services are designed for usage by non-IT experts.

The article is organized as follows. In Section 2 and Section 3, we present a review of data lake architecture focusing on the requirements that led us to choose a data lake architecture. In Section 4, we propose a design and an architecture for data storage with PostgreSql databases [41] and Simple Storage Service (S3) storage as main elements, with a full JavaScript Objet Notation (JSON) architecture for the IoT data. We will detail the design of data flows management from sensor networks by using the Elastic Stack for a near real-time visualization, the management of metadata by the implementation of a data catalog and the different tools to manage metadata and the design and implementation of the web site which is the entry and exit point for the end user of the data and metadata. In Section 5, different use cases will be shown. We conclude and propose some directions for future research.

## 2. Data Lake Architecture Review and Requirements

While it was still theoretical a few years ago, the concept of data lake used today in the academic and private sector has become a target of large infrastructure on a countrywide scale [42] or is evaluated in a large research infrastructure such as high energy physics and astronomy [43]. Data lake is also identified as a new trend in data management for agriculture [26].

Different definitions of the concept can be found in the literature over time.

The data lake concept has existed since 2010 [36] and is described as a large storage system for raw and heterogeneous data, fed by multiple data sources, and allows users to explore, extract and analyze all the existing data. A data lake has multiple synonyms now, such as data hub [44] or data reservoir [45]. Furthermore, the growing up of big data technical solution such as HADOOP [39,44] and more recently with proprietary solutions such as Azure [46], IBM [45] and Cloudera [47] have also introduced some confusion in people’s minds as they propose a fully technical operational solution with their own architecture.

Following the limitations of this concept highlighted in various scientific papers [39,45,48], Madera and Laurent [37] propose an enhanced definition to embrace scientific and vendors data lake definitions and pointed out three main challenges including Data Governance and Metadata Management and Enhancement.

Sawadogo et al. [49] modify Madera and Laurent’s definition of users (giving access to any type of user), of the logical view (modifying the handling of external data sources), and include scalability (which is a main requirements for a data lake for [36,50,51]). They also identify interesting features including indexing of data using direct or reverse indices, semantic enrichment and versioning of data.

Ravat and Zhao [52] propose to include input, process, output and data governance features in the data lake definition. They restrict access to the data lake to less users and include external data sources in the data lake (such as in Fang paper [39]). They make it clear that data ingestion is a process-free phase and that the raw data is stored in its native format for later use.

Sawadogo and Darmont in their review [53] adjust the definition and give new insights to manage and generate metadata. They also indicate that the management of unstructured datasets is not comprehensively globally thought, that the exploitation of unstructured data (such as text) is not taken into account, and that the possibility to cross-analyse data induces serious security problems.

Finally, Hai et al. [54] propose a new definition: “A data lake is a flexible and scalable data storage and management system that ingests and stores raw data from heterogeneous sources in its original format, and provides query processing and data analysis on the fly.” A data lake is not considered only as a storage system and must support on-demand data processing and querying. Furthermore, data indexing should only be performed if necessary at the time of data access, ingestion of data sources could be light, as there is no need to force schema definitions and mappings beforehand. Hai et al. agree that the metadata of the data lakes will evolve over time in an incremental way.

Whatever the definition, data lake can be simply defined as a schemaless architecture [50,55,56] using a schema-on-read global approach (even such a definition can be considered not precise enough for some computer scientists according to the literature given above), in contrast to the schema-on-write approach in warehouses.

Although the lack of a real consensus on data lake definition is true, we can resume that a data lake should keep data in its raw format and provides:

- Services to ingest any kind of data;
- Data access and use to various kind of users;
- A catalog service to make data findable by their metadata according to standards;
- A data governance;
- A logical and physical design;
- Some scalability possibilities.

When talking about data lake architectures, existing studies [52,57] generally distinguish between pond and zone or layer architectures.

Zone architectures generally consider three [58] to four zones [52] up to six zones [59].

In addition to this, several classifications of the data lake architecture have been implemented. Ravat and Zhao [52] propose to distinguish functional architecture from technical architecture while Sawadogo et al. [49] propose to classify the architectures into three groups (maturity-based, hybrid, functional). According to Sawadogo et al. [49], functional architectures include a data ingestion function to connect to data sources, a data storage function to hold raw and refined data, a data processing function, and a data access function to allow querying of raw and refined data. The components of data maturity-based architectures are defined according to the level of data refinement, while the components of hybrid architectures depend on both data lake functions and data refinement.

Giebler et al. [60] consider that defining data ingestion, storage and organization is not sufficient for a data lake, and then propose a framework based on nine aspects to define a complete data lake architecture not tailored to specific use cases.

Hai et al. [54] identify three examples of real applications of data lakes. Our project is of the type “multiple entries of heterogeneous raw data” with additions on:

- Managing geographic coordinates;
- Having a full JSON architecture for data querying;
- Considering a data valid for the infrastructure as soon as it has a “what, where, when” information;
- Combining relational and not only SQL (NoSQL) storage and querying systems;
- Giving the access to the data to any kind of users, including open data users;
- Furthermore, finally managing metadata with a catalogue managing spatially referenced resources.

## 3. The Elastic Stack

The Elastic Stack, initially designed to manage system log with a full text search approach is currently used to manage IoT data in several domains such as medicine [61,62], building monitoring [63]. The Elastic Stack [64] is composed of four main open and free projects: Beats, Logstash, Elasticsearch [65], and Kibana. Beats are data shippers. Logstash is a data processing pipeline. Elasticsearch is a document-oriented database. Kibana is a user interface tool of Elasticsearch for administration and queries. We are going present each tools in the next subsections.

### 3.1. Beats and Logstash

Beats are lightweight data shippers. They mainly ship data from the source, such as files, to Logstash. Logstash is a processing pipeline engine with real-time pipelines. It ingests data from multiple sources, and transforms and ships the transformed data to the configured destination, such as Elasticsearch.

### 3.2. Elasticsearch

Elasticsearch is the heart of the Elastic Stack. It is a free and open search engine with real-time and full-text searching. It is also a document-oriented and distributed database. The documents are JSON objects that are stored within an Elasticsearch index. An index field can be of many different types, e.g., a string or a number. The mapping of an index defines the types of the fields, and how the fields should be indexed and stored in Elasticsearch. Elasticsearch is a distributed database storage. It extends the concept of relationship between clusters, nodes, indexes and shards:

- A cluster can have one or several nodes.
- An Elasticsearch index can be divided into one or several shards (partitions).
- Shards of an index can be populated in one or several nodes in the same cluster.

One advantage of Elasticsearch in terms of storage is scalability and availability:

- Scalability: means that Elasticsearch can easily increase the size of storage, e.g., adding new nodes by configuring them in the same cluster, or adding new shards for an existing index without suspending this index.
- Reliability: means that if some nodes stop working, others in the same cluster can take the workload of the failing one.

Elasticsearch supports a varying number of field datatypes in a document [66]. There are core datatypes (e.g., string, numeric, date, boolean), complex datatypes (e.g., object for single JSON objects), geo datatypes, and specialized datatypes (e.g., Interne Protocol address).

Elasticsearch supports two groups of spatial data types which are of great interest for our platform: geo-point and geo-shape. Geo-point is a pair of latitude and longitude data. Geo-shape includes a point or set of points (e.g., point, linestring, polygon, multipoint, multilinestring, envelope, and circle).

Elasticsearch supports a query Domain Specific Language based on JSON data where a query structure combines three parts: Application Programming Interface (API) method; address that includes Uniform Resource Locator, index, type, and method; and the query body.

### 3.3. Kibana

Kibana [67] is an application used with Elasticsearch. An interesting visualization functionality of Kibana is the dashboard, as it supports Elasticsearch aggregation queries. We use Grafana instead of Kibana in the CEBA because it is more relevant for the main usages we have actually.

## 4. CEBA Architecture

Actual CEBA architecture has been designed according to the data flows it was funded to manage and the services it should give with the main hypothesis that it should tackle any data flow with no prerequisite.

The CEBA has been designed as a data lake with very few analysis functionalities as the main requirements were focused on data storage and sharing. According to the data management, the design is build upon the four main functionalities a data lake should provide on data (ingestion, storage, processing and access) with a metadata management at high level (at least, the datasets must have metadata).

As the CEBA can become in the future a big data platform for Auvergne Region, the storage layer has been designed to enable this (see Section 4.2, Section 4.3, Section 4.4) by using JSON as a common storage format.

Thus, the CEBA is composed of the following layers:

- An ingestion layer for streaming data sources using Beats;
- A dual data storage layer for near real-time visualization and data querying using Logstash, Elasticsearch, Grafana and PostgreSql;
- A long term data storage layer provided by PostgreSql and a S3 Mesocentre service;
- A data access and discovering layer by a website and a data catalog.

**Figure 2:**
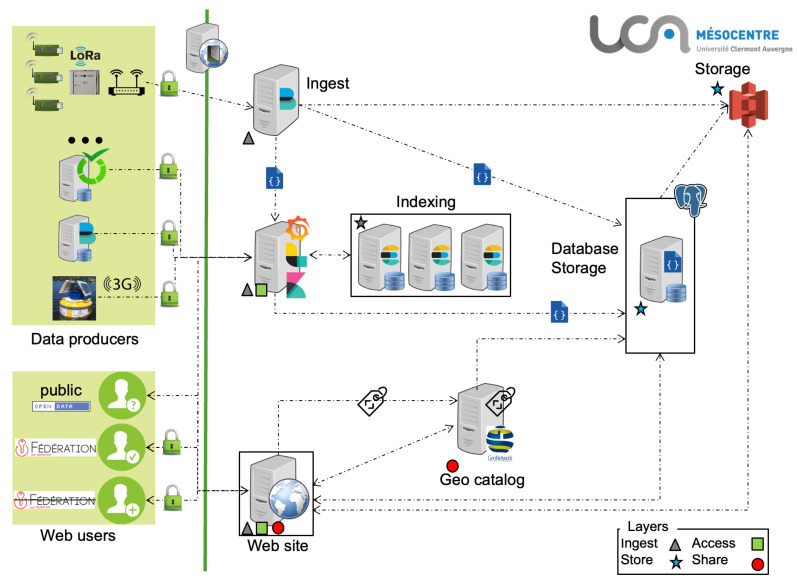
Global architecture of CEBA.

The CEBA architecture, displayed in Figure 2, is composed of several servers, each of them hosting one to several services. Each service is hosted by its own server. Specifically, the sensor network data part has one server for transport/transformation and a cluster of three Elasticsearch servers for indexing and retrieval.

**Figure 3:**
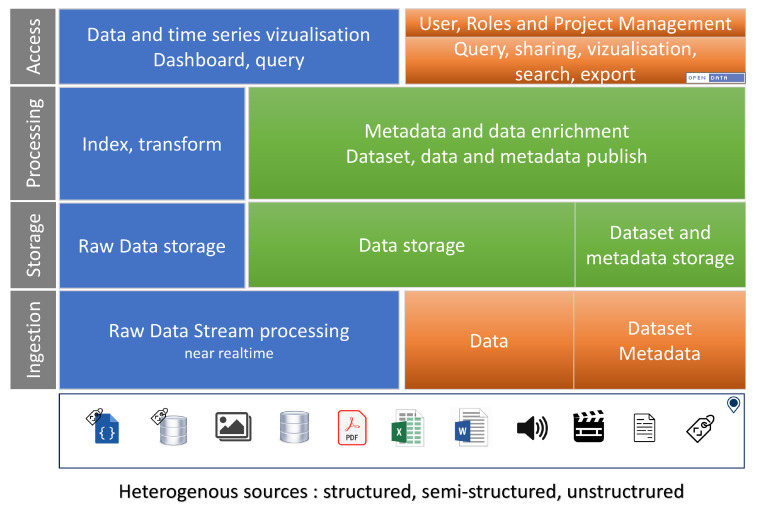
Global functionalities of CEBA (in blue IoT features, in orange web features and in green common features).

Regarding the functionalities, Figure 3 describes the different global functionalities of the CEBA, with the different possibilities of (i) depositing data and metadata, (ii) how they are stored with a unification of solutions, (iii) how they can be managed (indexing, enrichment, catalog), (iv) and finally how they can be manipulated and visualized. All features support at least GPS coordinates (GPS WSG84 latitude and longitude), even at the project level where the associated sites are described with a bounding box. Of course, all datasets and associated data can be made available in open data if they are declared as such.

The ConnecSens project from the European Regional Development Fund program of European Union (https://ec.europa.eu/regional_policy/en/funding/erdf/ (accessed on 1 December 2021)) has designed and implemented a collecting platform fully open source and proprietary, and is one of our main partner for the development of the IoT part [68].

In the next subsections, we are going to explain how data governance and management are set up and the design of several layers. We will start by describing the ingestion layer and the pipeline we set up. Then, we present the storage layer by describing the database and S3 components and how they are designed. We then finally describe the access layer composed of a web site and a the Grafana tool.

### 4.1. Data Governance and Management

Findable, Accessible, Interoperable, Reusable (FAIR) principles, open data and open science have driven data management as it was a clear issue to propose an open access to any data produced by public funds, applying the principle “as open as possible, as closed as necessary”. Data governance in the CEBA is not supported by a unique software solution but represented by good practices, requirements and choices for each software component. One main issue is the metadata management as any datafile to be uploaded must be associated with a dataset in order to make it findable and accessible on the CEBA, along with data coming from wireless sensor networks. As some partners are being part of Long Term Research infrastructures and national observatories such as INEE LTSER ZA (Long Term Socio-Ecological Research Zones Ateliers), OSU (Observatories of Sciences of the Universe) and OZCAR-RI (Critical Zone Observatories, applications et research) where data are mainly georeferenced, and linked to environmental studies, we are compliant with the Infrastructure for Spatial Information in the European Community (INSPIRE) directive. So, the metadata management is performed using INSPIRE [69], and more details are given in Section 4.1.1.

As multiple projects will use the CEBA platform and as the datafiles will be stored a quite long time (at least 5 years), a Data Management Plan (DMP) has been designed. It will also participate to the future Open Science initiative of University of Clermont Auvergne. This DMP model has been designed with the help of France Grilles Scientific Interest Group (www.france-grilles.fr/home/ (accessed on 1 December 2021)) staff and of INIST OPIDoR team (Optimiser le Partage et l’Interopérabilité des Données de la Recherche—Optimising Research Data Sharing and Interoperability). A draft of this model is being developed in the DMP OPIDoR platform, and needs to be finalized.

Considering the data stored, three cases can be considered: datafiles associated with datasets and data stored in the database, datafiles associated with wireless network and indexed data. For the first set, the life duration is the same as the dataset. They will be stored until the owner decide to delete it, and they will be accessible online anytime. For the second set, they will be stored until the owner decides to delete it, but are inaccessible unless specific use is needed. For the last set, we will consider a hot data subset and a cold data subset. They are distinguished by way they are accessible. Hot data are accessible via indexes, in near real-time meanwhile cold data are only accessible via database queries. The duration they are kept online, meaning accessible for visualization, is decided with the owner, and is generally adjusted to one year.

For dataset and datafile naming, we have proposed a naming convention with the main following elements: the producer identification, the production date, the geographical location of the data (city or region) and one or two words describing the data.

With regard to data governance, the choice was made to clearly distinguish between what data producers consider to be test and production streams. Although it may seem odd to keep test data, many CEBA users have stated that they have a real interest in keeping this data as it often contains good metrics. This way of distinguishing between test and production indices nevertheless allows many test indices to be removed to improve the performance of the elastic cluster.

For long-term resource management, the following organization was chosen: for one experiment, the current year’s data is stored in 12 monthly indexes to speed up indexing and querying, while the indexes from previous years will be merged into one by reindexing as we assume that the old data will need less frequent access.

#### 4.1.1. Metadata Management

We use Geometa [70] which provides facilities to manipulate geographic metadata defined with Open Geospatial Consoritum (OGC) ISO 19115 and 19139 eXxtensible Markup Language (XML) standards, to create representations of geographic metadata using R language, according the above standards, and write it in XML.

To ingest dataset’s metadata, we use the excel file designed by the collective project “Banking the Data Together” (BED) in charge of the data management within the Zones Ateliers network (RZA) (http://www.za-inee.org/en/node/804 (accessed on 1 December 2021)) and it enables the datasets ingest to respect the INSPIRE Directive [69]. RZA belongs to International Long Term Ecological Research network (ILTER) (https://www.ilter.network/ (accessed on 1 December 2021)).

As mentioned above, metadata and data are visible at three levels: public, private or embargoed.

- Open data: Anyone can access the metadata and associated data.
- Private: The metadata and data are only visible to members of the project. However, they are findable but not clickable from the search bar. If a user wishes to obtain more information, they should contact one of the metadata’s contacts.
- Embargo: Metadata is only visible to members of the project. It does not appear in a search. By default, they become open-data after one year.

Visibility also depends on how the metadata is deposited. By web form, it is possible to choose between these three modes, while by “drag and drop”, the games are automatically open data.

The metadata fields used in the CEBA are derived from the INSPIRE standard, in accordance with the FAIR principle. Some fields are mandatory in order to be compliant with the INSPIRE initiative (v1.3 currently) and to be as FAIR as possible in the first instance. We have twelve fields to describe the metadata itself (eight are mandatory), nineteen fields to describe the data (nine are mandatory), one mandatory field describing the quality of the data, one mandatory field describing the license for redistribution and reuse, and two optional fields describing access to the data. A contact (at least one author, one principal investigator and one point of contact are required) is described by fourteen mandatory fields. In terms of semantics, four fields can be used to semantically annotate the data. The first three are mandatory and correspond to INSPIRE topics, INSPIRE themes and GEneral Multilingual Environmental Thesaurus (GEMET) concepts that best describe the dataset. The fourth allows the use of any other semantic resource such as a controlled vocabulary, taxonomy, thesaurus or ontology (as long as the concept name and a Uniform Resource Identifier (URI) describing it are available).

Some optional fields are strongly recommended (such as geographic information and online access by URI to data) to be as FAIR as possible.

#### 4.1.2. Data Catalog

To make the available data visible and accessible, one solution is to use a data catalog that allows users to explore and understand their content through metadata. The goal is to store and redistribute data and metadata while respecting the FAIR principle (Findable, Accessible, Interoperable, Reusable).

After a comparison of several data catalogs (Dataverse https://dataverse.org/ (accessed on 1 December 2021), Ckan https://ckan.org/ (accessed on 1 December 2021), Zenodo https://zenodo.org/) (accessed on 1 December 2021), we chose GeoNetwork https://geonetwork-opensource.org/ (accessed on 1 December 2021), because it provides:

- The management of spatial, vector and grid referenced resources.
- Powerful editing and search functions for ISO 19115 or 19139 geographic standard.
- The management of metadata on reports or articles, with online access to digitized resources (e.g., Portable Document Format or via Digital Object Identifier (DOI)).
- Multilingual metadata editing, validation system (e.g., for INSPIRE recommendation) and geopublishing of layers in OGC services (e.g., GeoServer) exist.
- Harvesting from many sources, including: OGC Catalogue Services for the Web (CSW) 2.0.2 ISO profile, Open Archives Initiative Protocol for Metadata Harvesting (OAI-PMH) (http://www.openarchives.org/pmh/ (accessed on 1 December 2021)), Z39.50 protocols, web-accessible folders, ESRI GeoPortal and other GeoNetwork nodes.

Furthermore, it is currently used in many Spatial Data Infrastructure initiatives around the world, for example the ISRIC soil datahub (https://data.isric.org/geonetwork/srv/eng/catalog.search#/home (accessed on 1 December 2021)) or the FAO GeoNetwork (https://data.apps.fao.org/map/catalog/srv/eng/catalog.search#/home (accessed on 1 December 2021)).

This choice was confirmed after exchanges with the Centre Régional Auvergne-Rhône-Alpes de l’Information Géographique (https://www.craig.fr/ (accessed on 1 December 2021)).

The current production version in our infrastructure is the 3.8.1.0 version on a dedicated server.

### 4.2. Ingesting the Data

Data ingestion is the process of importing data from sources outside the system and storing it.

**Figure 4:**
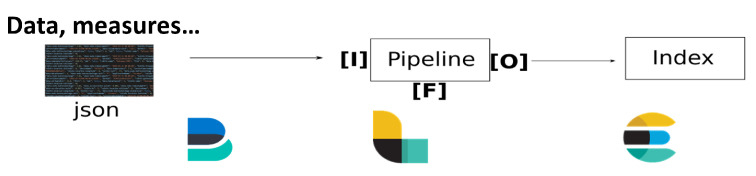
Schematic representation of generic ingestion pipeline.

For the sake of an instantaneous data visualization and to manage the different possible use cases, an ingestion multi-pipeline near real-time architecture has been designed in a generic way (see Figure 4). This pipeline is capable of handling data inputs of various sources: files, databases and data flow. For data coming from sensor network deployments, ingestion can be achieved by reading files created daily and fed continuously throughout the day. On the technical side, once the data file is created or updated with a new measure, a lightweight shipper, called Filebeat [64], forwards data to a data collector called Logstash [64]. This tool acts as a data streaming pipeline, that is, an Input–Filter–Output process that can ingest a multitude of data sources (Input), clean and enrich each event with some relevant information (Filter) and route data into the data lake (Output).

The routing of each measurement to the right storage location is conducted at indexing using an index naming convention in which the name of the experiment, the name of the node and finally the date of the measurement are used. This way, variables can be easily isolated and queried to generate time series.

This strategy offers the dual advantage of storing and retaining raw data through files and presenting it to the user in real-time through indexing.

Filebeat is a robust tool with capabilities to stop and resume data transmission in the event of a network failure and can slow down transmission in the event of ingestion problems with Logstash. Logstash, on the other hand, has data resilience capabilities through persistent queues that protect against data loss by storing each line of incoming data in an internal queue on disk. Together, these tools provide reliable, near real-time data management. Interestingly, it provides a visualization of the data allowing, for example, immediate verification of the installation of a sensor or node in situ.

Ingestion can also be performed using sources such as databases using a Java DataBase Connectivity plugin from Logstash, while Filebeat is able to ingest Message Queuing Telemetry Transport (MQTT) streams [71].

### 4.3. Storing the Data: Database

PostgreSQL is an opensource Database Management System (DBMS) versatile that allows to store a large number of different datatypes, frequently updated and has a very active community. It offers powerful tools (such as replication), being able to manage JSON data. However, as a relational DBMS, it lacks functionalities provided by NoSQL DBMSs to work on unstructured data. In addition, Postgres has multiple extensions such as Postgis [72] to handle and to query geographic data. The first paragraph details the database design, then we explain how dataflows are ingested in the database. Then the proposed database schema is given, on which we then detail the data enrichment process, the stored queries and the data safety.

#### 4.3.1. Database Design

The sensor database is structured around the associated sensor networks. Several schemas have been set up according to the structure of data received. We currently have two schemas: one for delimited files and one for ConnecSens network with JSON files. Since JSON is the chosen format for IoT data management in our platform, so it is in our database. It is thus sometimes necessary to transform raw data files into JSON files.

Within PostgreSQL, JSON and JavaScript Objet Notation Binaries (JSONB) are available to store the raw JSON data. We chose JSONB which currently has more query and comparison tools than JSON. JSONB combines the flexibility of NoSQL solution with the querying power of Relational Database Management System (direct query of the JSON attributes contained, such as listing values or listing keys, comparisons, selection between 2 dates…). However, inserting JSONB into a database takes a little bit longer than inserting JSON due to the conversion. It is also possible to create indexes on attributes of a JSONB row which improve the speed of repetitive queries. Between Binary Tree (BTREE), HASH and Generalized Inverted Index (GIN) index types we have chosen BTREE because it is the most commonly used index in relational databases and the lightest, comparison operations are faster when associated with a key-value pair, unlike the HASH index which is more rigid. We may move towards GIN indexes if the queries to be provided become more complex as it is heavier but offers additional search and comparison operators in JSON, as well as speeding up searches such as a classical index. Using JSONB has other advantages because, unlike traditional JSON which stores its data in plain text, JSONB stores the data in a decomposed binary form. This makes query processing more efficient and faster.

As each sensor network sends JSON lines configured differently, each schema has its own indexes.

#### 4.3.2. Database Ingestion

**Figure 5:**
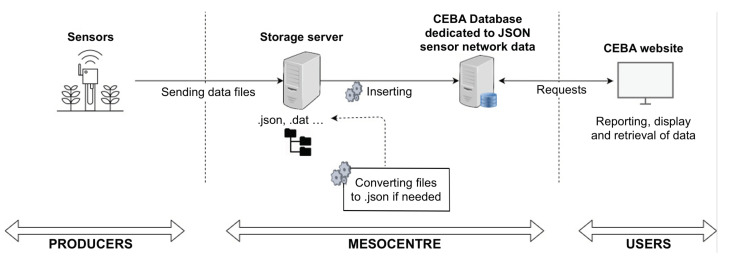
Dataflow for sensors database ingestion.

Forgetting the authentication steps, all the data coming from sensor networks reach on our storage servers located at the Mesocentre in Clermont-Ferrand, and are inserted into the database via a daily running script to insure that the data stored into the database is correct (see Figure 5).

If the data received is not in JSON, another script transforms it. Once the data is in the database, it is then possible to query it from the web site by the CEBA users.

#### 4.3.3. Database Schema

**Figure 6:**
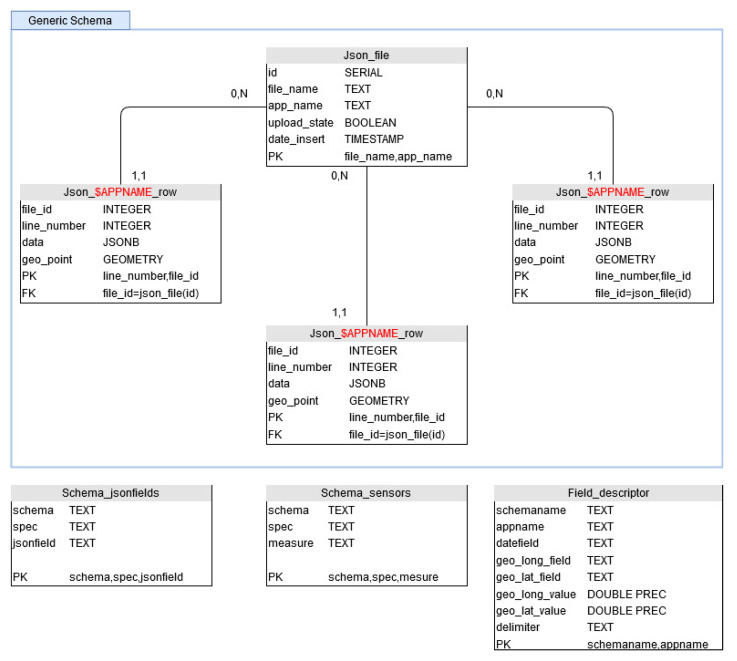
Schema of the database.

As the data we received are schemaless, we have designed the database in a generic way, in order to be able to store, isolate and query easily any sensor network. We have chosen to use PostgreSQL schema to represent a type of sensor network (Figure 6). Within a schema, we find sensor networks having this same type. Actually, we are managing semi-structured JSON format and delimited files. In order to distinguish them, we give them an application name ($APPNAME). Each deployed sensor network then has its own storage table. It is possible to have as many tables as we want within a schema. As sensors networks can be modified during the time, we can follow all the changes this way, as a new table can be added whenever it is needed to not mixed data coming from various deployments. In order to insure data management, the file name and the insertion date are stored (see table Json_file in Figure 6). With this information, it is possible to quickly identify data transmission or data corruption problems.

#### 4.3.4. Data Enrichment

**Figure 7:**
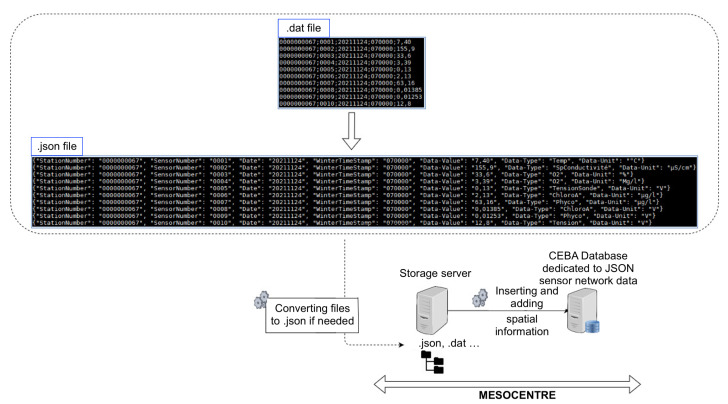
Converting and enrichment flow.

Sometimes, sensor networks do not return enough information to be properly queried. In this case, complementary information tables are filled with additional data such as GPS coordinates (in case of static deployment), the list of sensors or the list of measurement types. This allows to enrich the data inserted in the database during the retrieval and to allow a more efficient querying. For example, a network of sensors is linked to a buoy on the Lake of Aydat, autonomous in energy. This system sends very small delimited files with no header by text message, without GPS coordinates (GPS WSG84 latitude and longitude) or information on the type of data recovered or the unit. We have then automated two tasks for this network: transforming the received files into .json files before inserting them into the database, as well as enriching the information during this same transformation with sensors names, units and GPS coordinates. This is conducted by using rows of tables Schema_jsonfields, Schema_sensors and Field_descriptors in Figure 7.

#### 4.3.5. Stored Queries

For the user side on the web site, stored queries have been designed and implemented on the database server side. These queries are “generic” ones, as they can query any JSONB content of any sensor network (as the database structure has been design to enable this). However, the number of fields returned differs depending on which has been transmitted. As a result, more accurate and powerful queries have been implemented for ConnecSens’ sensor networks since the network returns a lot of information.

**Figure 8:**
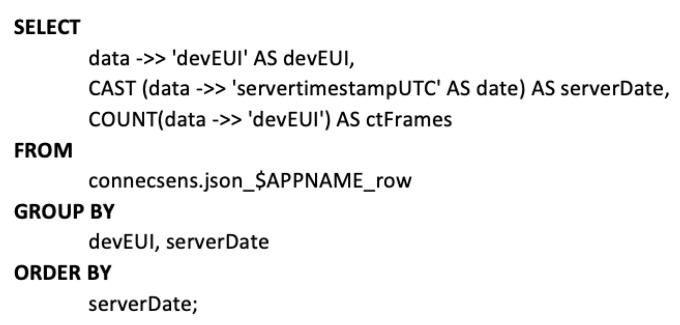
Stored query example.

For example, Figure 8 shows a query to display the evolution of the number of frames per node (DevEUI) per day, for a specific $APPNAME.

The “->>” sign allows one to access a specific key in the JSON and to obtain values. It is possible to cast its type or to perform tests on the value, such as any PostgreSQL attribute. To understand this request, here is some information on how a JSON file is generated from a ConnecSens sensor network. Each JSON line contains a “DevEUI” key. Counting this key is a way to count every frame sent. Then, casting the “ServerTimestampUTC” key to the date format allows one to obtain only the date without the time.

Such a simple request has two types of exploitation: a technical exploitation because it allows the rapid identification of transmission or power supply problems, and a scientific exploitation because if the transmission is linked to a particular expected event, it can allow the rapid detection of the appearance of a phenomenon to be studied.

#### 4.3.6. Data Safety

Several types of data backup are performed. The Postgres database, which includes data from the website and sensor network, is replicated in real-time on a second server, which identically reproduces the transactions that take place on the main server. The entire server is backed up every day in order to be able to perform full restorations. Finally, the data of the sensor network database and the structure of the tables are exported and archived in the S3 every week.

### 4.4. Storing the Datafiles: S3

To store the datafiles within the CEBA, we use a local session of an S3 provided by the Mesocentre. On the backup side, the data deposited in a bucket is replicated three times.

We have chosen S3 as it is a solid storage solution because:

- It is scalable because there is no fixed limit in terms of storage capacity or data transfer.
- The versioning function allows one to store several versions of the same object in the same Bucket.
- It is secure thanks to the numerous encryption and data management functions. On the server side, encryption can be performed with three key management options and client side encryption.
- Due to its low latency and high throughput, S3 Standard is suitable for dynamic websites, content distribution and Big Data analysis.

### 4.5. Accessing the Data

Allowing different users to deposit, discover, access, visualize, extract and query data are part of the functionalities that a data lake must offer (see Section 2), while guaranteeing a sufficient level of security. The design of the corresponding tools and services must also ensure a scientific exploitation of the data by their producers while ensuring a sufficient level of description via metadata to allow scientific discovery and reuse of the data.

To this end, we have designed a single entry point for the user in the form of a website, and a real-time consultation service for sensor network data using the Grafana tool. These two access points are interconnected, secure, and provide access to all data, regardless of format and storage.

We will now detail the proposed website in terms of its operation and design. Then we will describe the configurations made on Grafana to ensure the real-time visualization of the sensor network data.

#### 4.5.1. Web Site

In the following, we will first describe the design and security, the project-based structuring of the data repository and management, the management of the metadata repository and access, and finally the access to the sensor network data.

##### Symfony Update and Security

The website is developed with the Symfony framework (https://symfony.com/ (accessed on 1 December 2021)) version 4.4; this version is checking security patches until November 2023. Moreover, the updates of the framework are made fluid thanks to the notion of “future compatible”. In terms of security, Symfony provides many tools to secure the application: secure session cookies by default in Hypertext Transfer Protocol (HTTP), Cross-Site Request Forgery (CSRF) protection.

##### Testing

To perform tests, we used standard tools such as the PHPUnit framework (https://phpunit.de/ (accessed on 1 December 2021)) for unit tests and also OWASP Zed Attack Proxy (https://owasp.org/www-project-zap/ (accessed on 1 December 2021)) to test the software security.

##### Deployment

In order to easily deploy and maintain the CEBA web solution, some parts of the CEBA such as R server are docked using Docker (see Figure 2) and the source code of the website is stored, backed up and versioned thanks to the code management software “Git” on a dedicated space of the university.

##### Security Aspects

We have used the Fédération Éducation-Recherche (a service provided by Groupement d’Intérêt Public RENATER (https://www.renater.fr/ (accessed on 1 December 2021)) to enable a trusted connection to our website in order to foster nationwide collaborations. At first, we installed and configured the Shibboleth software (https://www.shibboleth.net/ (accessed on 1 December 2021)) to connect the CEBA website to the federation of authentication authorities. As no version of Shibboleth was implemented with the version of the Symfony Framework we used, we then developed and validated the interconnection of these services. If the user is not a member of a trusted organization, we can create a local account. Examples of local accounts are the Chamber of Agriculture or private partners.

##### Users Management

The CEBA site offers three levels of roles for users: “super administrator” reserved only for CEBA computer specialists, “administrator” to manage projects and “user” to allow connection but has no rights by default. Unlike public access, the “user” role allows you to be included in a project and to have full access to the associated resources.

##### Project Structure

The steering committee asked for the website to be structured structured around the notion of “project”. This notion has been broken down into two levels: structuring and standard. A project can be “structuring”, i.e., it can contain other projects or not.

A project is defined by a name, a status, a funding, a creation date, a supporting organization, a website (by default the organization’s website), whether it is of a ConnecSens nature or not, an image, a summary, a description, a project owner, affiliated users or a geographical site, whether it is structuring or not. A project is created by a user with the administrator role. A project contains the following headings: experiment, site, sensor network and dataset.

An experiment is characterized by an instrument, a study object and a method in a project and can be linked to several sites.

A site is a geographic location defined by a longitude, latitude and a name to identify the location of the project.

Sensor networks of any type can be connected to the project (see Section 4.2, Section 4.3 and Section 4.5.2 for more details).

Finally, datasets can be created in two ways: by form (unitary deposit), or by “drag and drop” from a tabular file to the INSPIRE standard (dataset deposit) Section 4.1.1. In order to properly manage the life cycle of metadata and data, it is possible to restrict the visibility of a dataset by applying a “public”, “private” or “embargoed” status. The dataset file can be downloaded in XML format. Once the dataset is created on the CEBA, a data file can be uploaded in any format with a limit of three gigabytes and associated with the dataset. It is stored on the S3 Mesocentre. A web feature checks that the data file names are unique to avoid accidentally overwriting an old file. Deleting a data file on the website implies a permanent deletion on the S3.

##### Project Management Rights

It is possible to distribute the authorizations to edit the different parts of the project between the users affiliated (user role) to a project. A user can be assigned from zero to several permissions by the project owner to manage (Create, Edit, Delete) the associated part of the project. Five categories have been created: project management, sensor network management, create an experiment, declare a site, declare datasets.

The Project Management permission (grant the same rights as project administrator on the project data) allows one to edit a project, manage all the metadata related to the project, manage the sensor networks and give rights to the project users. The Manage Sensor Network permission allows one to connect a sensor network to the project. The Create Experiment permission allows one to add experiments to the project. The Declare Site permission allows one to add a geographic site already entered in the CEBA to the project. The Declare Datasets permission allows one to deposit datasets into the project and manage them by modifying or deleting them.

All user permissions in a project are summarized in a table located in the project and visible only to the project owner or administrator.

##### Data Catalog Flows

**Figure 9:**
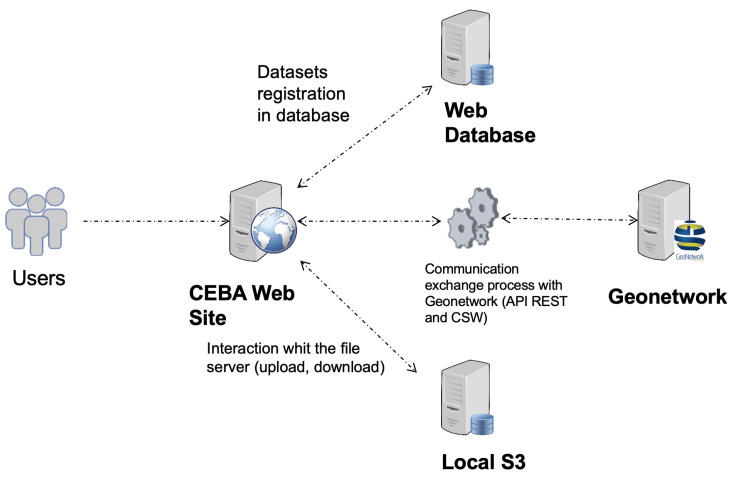
Flows between website and data catalog.

For security reasons, the data catalog is not publicly exposed. The website and GeoNetwork communicate (see Figure 9) with two technologies: Representational State Transfer (REST) API provided by Geonetwork, CSW protocol. The REST API allows us to view/delete datasets from our website for the queries that do not require an extraction or an XML file. The second one, the CSW protocol, allows to expose catalog records via HTTP.

We are now going to detail the creation, edition deletion and visualization of a dataset.

To deposit and therefore create a dataset from the website, all the variables of the form (web or excel) are retrieved and two Comma-separated values (CSV) files are created. The first one contains all the metadata information while the second one contains the contact information (by drag and drop, the information is already in CSV format). These CSVs are injected into our processing chain consisting of an R tool called Geometa [70] which creates an XML from the two tabular files, using the ISO 19115 standard. Then the XML is sent via the CSW 2.0 protocol to the GeoNetwork. Once the process is finished, the status of the operation is retrieved to know if there was an error or not and to display the result.

To edit a dataset, a request via the REST API is used to retrieve the metadata record of the dataset, then the previous processing chain is used. It is possible to add data files to the form, which are saved on the S3 in buckets dedicated to the project where the dataset is located.

The dataset deletion is also performed by request with the REST API according to the same principle. All the resources (form and associated data files) are then deleted.

The REST API can be used to obtain the desired form in ISO 19115 XML format, which is then displayed on the website.

##### Searching into the Metadata

As requested by the steering committee, the CEBA website has a metadata search function powered by a search bar. Currently based on an Structured Query Language query, it returns open data datasets (ignoring embargoed datasets, and private datasets are returned but remain inaccessible) by searching multiple fields (title, abstract, gemet_keywords, INSPIRE_keywords, other_keywords, and lineage). In the future, we plan to improve our search capabilities by dedicating a web page and an API to search the index. In addition, it will be possible to search by date, area (geographic query), resource type, status, format and representation type, such as sensor network data.

##### Sensor Network Access

From the website, depending on the richness of the data transmitted (at least “what”, “when”, “where” in a native or enriched way), it is possible to execute temporal or spatial queries, or even complete extractions of more or less complete raw data downloadable in json or CSV format (see Section 4.3).

The user will be able, according to his rights in the CEBA and in the project, to access the Grafana interface to consult the data in real-time on the Grafana dashboards (see Section 4.5.2).

#### 4.5.2. Grafana

Data visualization is conducted through time series generated by queries on quantities (variables). From the CEBA side, we only provide a data source with which users (scientists and IT) can design their own dashboard. It is striking to note that the simplicity of the queries allows all users to create their own graphs. The secure access through the use of a personal account and the Hypertext Transfer Protocol Secure protocol allows to isolate the data, i.e., to provide each user with access only to the data he/she needs and is authorized to access. This is possible thanks to the combined use of group and organization with the data source in Grafana. An interesting point is the ability to create data sources for particular events by reindexing existing data reduced to defined variables, time interval or material. This reindexed source can then be deleted once the event is over. Finally, although focused on the real-time aspect, dashboards can also be used for more sophisticated queries, such as cross-referencing or historical data. In this case, these charts retrieve data from sources built on PostgreSQL databases.

## 5. CEBA in Action

In this section, the use of the CEBA for several projects is shown, and some numbers of CEBA data hosting are given.

### 5.1. Etna Use Case

**Figure 10:**
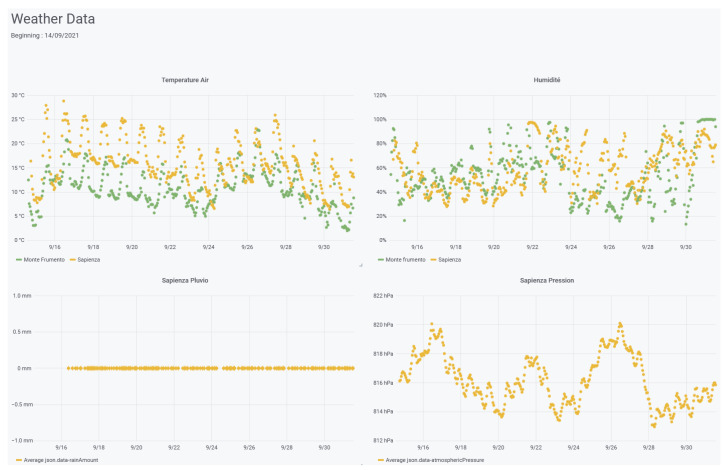
Extract of the dashboard of weather station at Mt. Etna (courtesy of LPC and LMV).

The Laboratoire de Physique de Clermont (LPC) and the Laboratoire Magmas et Volcans (LMV) of the University of Clermont Auvergne have led a scientific project to continuously monitor atmospheric radon on Etna in 2019. In parallel with the development of new radon probes, they used the ConnecSens project technology to deploy a network of sensors. Prior to on-site deployment, autonomy and data transfer were tested to validate the design and configure data transmission. The CEBA was involved in this step to find the right ratio between energy consumption, management and data analysis. During the deployment on Etna, the CEBA was very helpful as people on the volcano were able to check the data transmitted in real-time on their smartphones, and thus validated the deployment and configuration of the wireless network within a week. Since 2019, in parallel with the different deployments and with the addition of new sensors, the collected data are stored in the CEBA. For project users, real-time data is displayed and accessible via grafana, and data can be queried from the website (see Figure 10). Monitoring the wireless network is also very useful because it allows the IT staff to verify that it is functioning properly and to prepare upgrade and repair tasks for the next mission on Etna. In this case, the CEBA was the key technology point that enabled real-time aerial radon monitoring on Etna for the first time [68].

### 5.2. ZATU Use Case

The CEBA can be used to share, make visible and store the analysis and results obtained by a science project. ZATU has been a member of the French LTSER network (Réseau des Zones-Ateliers) since January 2015, and this long-term socio-ecological research observatory studies the relationships between ecology, society and radioactivity. The ZATU LTER observatory is located in a geographic area northeast of Clermont-Ferrand on the border of the Limagne Valley and the Bourbonnais Mountains in France. The members are from twenty-two laboratories or institutes, producing numerous data from sampling, analysis, models and results on soil, air and water. The data come from the three main themes focused on humanities and social sciences, radio- and geochemistry, and biology and ecology, respectively. ZATU uses the CEBA for two primary purposes: data repository and dataset catalog. ZATU users share the data they are working on through the data catalog and storage features. ZATU data managers have created several datasets, to which they are currently adding the datafiles to be shared. This makes the data produced by the members and the projects around the ZATU visible, accessible, and findable. One of ZATU’s projects was to design a stand-alone wireless radon sensor. In order to test the quality of measurements, data transmission and autonomy, one of these systems has been deployed on the university campus and is currently sending data. The CEBA assisted in the discussions and validation of the prototype designed in collaboration with a company. In early 2022, the observatory will be equipped with several wireless sensor networks measuring many variables such as radon in the air, water level and flow, and weather that will be uploaded and stored in the CEBA.

### 5.3. Aydat Monitoring

Aydat lake, located in the French Massif Central, has a total area of 60 hectares, a small watershed of around 16.8 hectares and a maximum depth of 15 m. It is a eutrophic lake with recurrent proliferation of cyanobacteria. This lake belongs to the “Observatoire des Lacs” (OLA) network which contributes largely to research on lake ecosystems in France. The research programs concern the global understanding of the hydro-ecological processes of the lake in order to better predict its responses to current and future changes, with the aim of monitoring and reducing the proliferation of cyanobacteria (which has a great impact on the local economy, on the functioning of ecosystems and reduces biodiversity). Three main types of sensors systems are deployed on the lake (one HYDROLAB HL7 multiparameter sonde, three Aquatroll 200 data logger, eight HOBO temperature data loggers) with different transmission technologies (LoRa using ConnecSens technology with a gateway connected to the internet for Aquatroll, Short Message Service for HYDROLAB HL7 and Bluetooth for HOBO data loggers) sending at least datapoint hourly, each managed by different projects. HYDROLAB HL7 multiparameter sonde comprises eight sensors (a conductivity sensor, Hach LDO® Dissolved Oxygen Sensor, temperature sensor, turbidity Sensor, chlorophyll-a sensor, blue and green algae sensor, rhodamine sensor, and finally a pressure sensor for water depth measurement). For the Aquatroll, the water level measurement is based on a piezoresistive sensor whereas the water temperature and the specific conductivity, the salinity and the total dissolved solids (TDS) is monitored using a balanced 4-electrode cell. The HOBO data loggers record temperature with high-frequency acquisition (i.e., 5 min). The Aquatroll and HYDROLAB HL7 systems are currently sending their data hourly to the CEBA, and reference datasets have been created by the projects around this observatory.

The CEBA allows all partners to access all data produced (in real-time, on historical data and on analysis).

**Figure 11:**
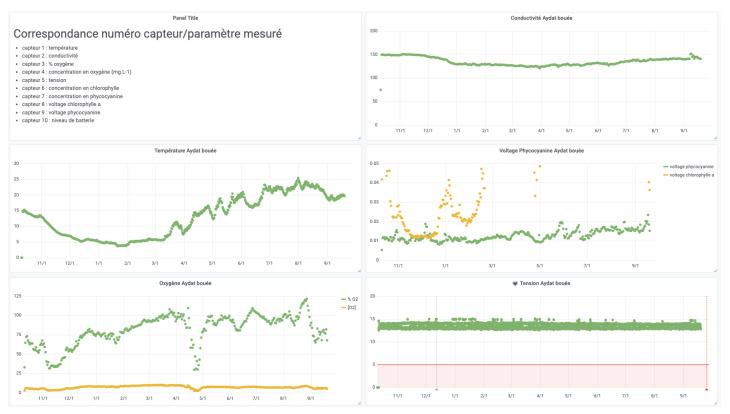
Extract of the dashboard of one system—courtesy of Aydat Observatory.

For data visualization, dedicated interfaces have been built for each data collection project (see Figure 11 for example). A project can range from a group of sensors in the same area or in different areas to a specific monitoring site.

These interfaces allow to compare the measurements of several nodes equipped with the same sensors. This makes it possible to compare different areas or experimental deployment conditions (using a graph for a quick overview or a table for a more complete analysis).

**Figure 12:**
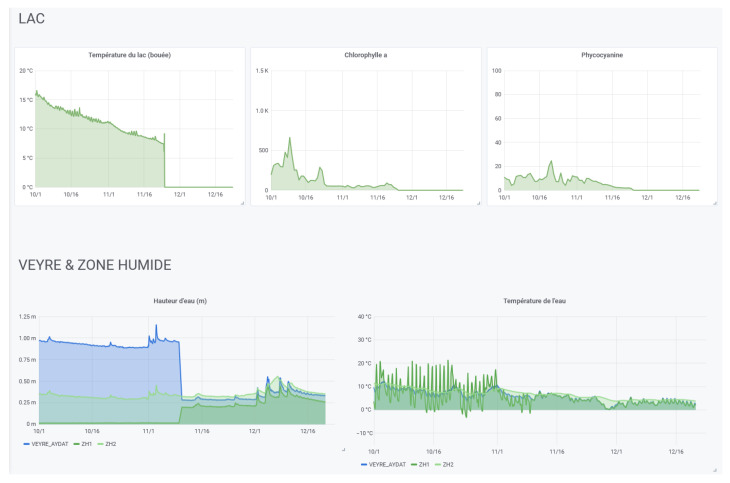
Extract of the dashboard of multiple independent sources—courtesy of Aydat Observatory.

We also demonstrated that by using the features of Elasticsearch and Grafana explained in Section 4.5.2 (notably with the ability to reindex), with the agreement of the owners, the CEBA is able to give access to independent data sources (Elasticsearch indexes or PostgreSql tables) to gather all the data produced on the same geographical site or on the same theme (see Figure 12).

### 5.4. CEBA in Numbers

Eleven projects are hosted by CEBA website, but several projects are under construction. Nine wireless sensor networks are sending data, some for more than three years, for around five millions records in the database and more than four millions in the elastic cluster. All these records weight from 3 GB to 5 GB in the database and in the elastic cluster, respectively. More details are given in Table 1 and Table 2. The number of records has increased from a factor of 4.5 between 2020 and 2021. In the same time, the indexing duration has been kept good going from 0.9 ms in 2020 to 1.5 ms in 2021. The query duration is 0.3 ms for the current use. These figures must be taken with care because they take into account the various evolutions carried out on the cluster such as the limitation to one year of hot data in the indexes is conducted to optimize the performances.

## 6. Conclusions

This paper presents proposals and operational responses to a set of requirements arising from the CEBA project, which led to the design and implementation of an eponymous platform.

The first requirement is to manage geographic coordinates throughout the data management process, from ingestion to visualization and querying. This has been achieved by implementing various elements such as a catalog to manage spatially referenced resources with Geonetwork, a database storing geographically extended data in JSONB using PostgreSQL and the PostGIS extension. Data enrichment during ingestion, when GPS coordinates cannot be transmitted, is performed both in the database and in the indexing pipeline. This allows hot and cold data to be queried in the database and index with almost the same complexity as geographic queries. From a normative point of view, the ISO 19115 and 19139 standards are managed and the INSPIRE directive is used, to be compatible with other infrastructures.

Secondly, the “what, where, when” information was needed. For this purpose, the corresponding metadata are managed in all components of the platform and can be queried, allowing data and datasets to be found using this information.

Third, to manage the ingestion of any kind of data stream from IoT networks, we proposed and designed an ingestion pipeline based on the Elastic Stack. This pipeline feeds an Elasticsearch cluster providing near real-time data access, which in our experience has proven to be scalable and responsive to the increasing amount of data.

Fourth, the entire storage and indexing infrastructure was designed in JSON to facilitate data querying and enriching (especially from sensor networks). This functionality allowed data that would normally be isolated from each other to be retrieved and queried on demand.

Finally, in terms of additional functionality, we proposed and designed a unique data management system in the form of a website that allows data to be uploaded, downloaded, queried and viewed. It also allows for the creation and management of datasets for which data files can be deposited. Users can thus use a single tool for open data purposes and for managing the data produced by scientific projects with secured access.

This reliability of the CEBA platform has been validated by several projects such as the monitoring of radon in the air on Mt Etna [68], the global monitoring of Aydat Lake and some new collaborations with industry. CEBA also hosts the datasets and data files of long-term research infrastructures and their projects, such as the ZATU workshop.

Currently only available in French, an English option will be added in the next version of the web site.

In order to improve the query and analysis capabilities of the CEBA, which are currently quite limited and to reach a level similar to that of data warehouses, research is underway [73] to build a query engine on top of the CEBA (with Elastic Stack or Apache Spark SQL [74]). The focus will be on spatial queries with time series criteria. In order to be able to tackle the IoT network at higher speeds, we are testing other stream processing platforms such as Apache Kafka [75] and Apache Spark.

## Figures and Tables

**Table 1:** Table describing the number of records and the size in the database.

Project	Records	Size (in GB)
ConnecSens	3.0 M	3.8
Aydat	0.4 M	0.6
Etna	0.7 M	0.8
ZATU	0.3 M	0.3
Total	>5 M	>6

**Table 2:** Table describing the number of records and the size in elastic cluster.

Project	Records	Indexe Size (in GB)	Indexes
ConnecSens	2.4 M	2.2	302
Aydat	0.7 M	0.7	62
Etna	0.6 M	0.56	78
ZATU	0.4 M	0.4	37
Total	>4.5 M	3	>500

## Data Availability

No new data were created or analyzed in this study. Publicly available datasets shown in this paper can be found here: https://ceba.uca.fr. Some data presented in this study are available on request from the corresponding author.

## Abbreviations

The following abbreviations are used in this manuscript:

API	Application Programming Interface
BTREE	Binary Tree
CEBA	Cloud Environnemental au Bénéfice de l’Agriculture
	Environmental Cloud for the Benefit of Agriculture
CSRF	Cross-Site Request Forgery
CSV	Comma-separated values
CSW	Catalogue Services for the Web
DBMS	Database Management System
DMP	Data Management Plan
DOI	Digital Object Identifier
FAIR	Findable, Accessible, Interoperable, Reusable
GEMET	GEneral Multilingual Environmental Thesaurus
GIN	Generalized Inverted Index
HTTP	Hypertext Transfert Protocol
I-Site	Initiatives Science–Innovation–Territoires–Economie
	Initiatives Science–Innovation–Territories–Economy
INSPIRE	Infrastructure for Spatial Information in the European Community
IoT	Internet of Things
IT	Information technology
JSON	JavaScript Objet Notation
JSONB	JavaScript Objet Notation Binaries
LoRa	Long Range
MQTT	Message Queuing Telemetry Transport
NoSQL	not only SQL
OAI-PMH	Open Archives Initiative Protocol for Metadata Harvesting
OGC	Open Geospatial Consoritum
REST	Representational State Transfer
S3	Simple Storage Service
URI	Uniform Resource Identifier
XML	eXtensible Markup Language
